# Comparison of Access Site Complications in Primary Percutaneous Coronary Intervention (PCI) Using the Radial Versus the Femoral Approach for Complex Lesions: A Prospective Study

**DOI:** 10.7759/cureus.72781

**Published:** 2024-10-31

**Authors:** Fraz Ahmad, Ahmad Usman, Unknown Osama, Aneela Afreen, Hafiz Mian Muhammad Farhan, Shohaib Daniyal, Sadiqa Jamil, Fahad R Khan

**Affiliations:** 1 Cardiology, Shalamar Medical and Dental College, Lahore, PAK; 2 Cardiology, Army Cardiac Centre, Combined Military Hospital, Lahore, PAK; 3 Internal Medicine, Army Cardiac Centre, Combined Military Hospital, Lahore, PAK; 4 Cardiology/Internal Medicine, Army Cardiac Centre, Combined Military Hospital, Lahore, PAK; 5 Cardiology, Lady Reading Hospital, Peshawar, PAK; 6 Interventional Cardiology, Peshawar Institute of Cardiology, Peshawar, PAK

**Keywords:** access site complications, complex coronary lesions, femoral access, percutaneous coronary intervention, radial access, st-elevation myocardial infarction

## Abstract

Background: Percutaneous coronary intervention (PCI) is a widely used therapeutic approach for complex coronary artery disease, especially in patients with ST-elevation myocardial infarction (STEMI). The choice of vascular access site, typically radial or femoral, can significantly impact patient outcomes due to varying complication rates associated with each approach.

Objective: This study aimed to compare access site complications between radial and femoral approaches in primary PCI for complex coronary lesions, providing insights into the safety and efficacy of these approaches.

Methods: A prospective cohort study was conducted from January 1, 2023, to December 31, 2023, at a tertiary care cardiovascular center. A total of 350 adult patients presenting with STEMI and requiring emergency PCI for complex coronary lesions were included and randomized equally to either radial (n = 175) or femoral (n = 175) access groups. Primary outcomes included access site complications, such as hematomas, pseudoaneurysms, arteriovenous fistulas, and major bleeding events. Secondary outcomes included procedural success, access site crossover, and hospital stay duration. Data were analyzed using chi-square tests, Student's t-tests, and multivariate logistic regression.

Results: The incidence of access site complications was significantly lower in the radial group (11.4%) compared to the femoral group (22.9%) (p = 0.007). Major bleeding events were also notably reduced in the radial group (2.3% vs. 8.6%, p = 0.01). There was a significantly shorter median hospital stay for the radial group (three days vs. five days, p < 0.001), while procedural success rates were comparable between groups (97.1% vs. 94.3%, p = 0.31).

Conclusion: The radial approach for primary PCI in complex lesions is associated with fewer access site complications and shorter hospital stays compared to the femoral approach, supporting its use as the preferred access site.

## Introduction

Percutaneous coronary intervention (PCI) is a widely adopted therapeutic approach for the treatment of complex coronary artery disease, particularly in patients presenting with ST-elevation myocardial infarction (STEMI) [1]. Over the past few decades, PCI has significantly evolved, leading to improved procedural success rates and reduced mortality. The choice of vascular access site plays a crucial role in determining patient outcomes, with two main sites commonly utilized: the radial and femoral arteries. Access site complications are an important consideration during PCI, especially for complex coronary lesions where the complication rates can vary significantly depending on the access route employed [2].

Traditionally, the femoral artery has been the preferred access site due to its larger vessel size, which facilitates the use of larger catheters and devices [3]. However, it is associated with a higher incidence of vascular complications, such as hematomas, pseudoaneurysms, arteriovenous fistulas, and major bleeding [4]. In contrast, the radial approach has gained popularity in recent years due to its association with reduced complication rates, quicker recovery times, and improved patient comfort [5]. Numerous studies have demonstrated that radial access is associated with fewer access site complications and shorter hospital stays compared to femoral access [6].

Despite these advantages, the use of radial access for PCI in complex coronary lesions remains a subject of debate due to procedural challenges. Complex PCI often involves longer procedural times, intricate lesion characteristics (such as calcified or bifurcated lesions), and a greater need for catheter stability, factors that traditionally favor femoral access. Additionally, anatomical challenges such as small radial artery diameter, severe radial artery tortuosity, or previous occlusions may necessitate switching to femoral access [7]. Previous studies have predominantly focused on general populations undergoing PCI, often overlooking the unique challenges posed by complex lesions [8].

The present study was designed to compare access site complications between radial and femoral approaches in patients undergoing primary PCI for complex coronary lesions. By focusing on this high-risk subgroup, this study aims to provide more precise data regarding the safety and efficacy of these approaches. The findings could have significant implications for clinical practice, particularly in optimizing access site selection for high-risk PCI cases.

## Materials and methods

Study design and duration

This prospective cohort study was conducted between January 1, 2023, and December 31, 2023, with the aim of comparing access site complications in patients undergoing primary PCI for complex lesions using either radial or femoral approaches. The prospective design allowed for the observation of real-time clinical outcomes, reducing recall bias and enabling direct assessment of intervention-related complications over time. Ethical approval was obtained from the Institutional Review Board (IRB), and the study was conducted in accordance with the Declaration of Helsinki. Written informed consent was obtained from all participants.

Setting and participants

The study took place at the Army Cardiac Centre, Combined Military Hospital (CMH), Lahore, a tertiary care cardiovascular center. Participants were recruited according to strict inclusion and exclusion criteria to ensure a representative sample of patients undergoing primary PCI for complex coronary lesions.

Inclusion criteria: Adults aged 18 years or older presenting with STEMI requiring emergency PCI for complex coronary lesions as identified by angiography and patients eligible for either radial or femoral access were included.

Exclusion criteria: Patients with contraindications to either radial or femoral access, significant comorbidities affecting vascular healing, end-stage renal disease, prior coronary artery bypass grafting, or inability to provide informed consent were excluded.

Sample size calculation

The sample size for this study was calculated using the World Health Organization (WHO) sample size calculator, accounting for the prevalence of access site complications in similar populations. Based on an estimated prevalence of 12% for access site complications in patients undergoing complex PCI using the femoral approach, with a confidence interval of 95%, power of 80%, and an effect size suitable for detecting significant differences between groups, and the required sample size was 350 participants. These participants were randomized equally into two groups (radial, n=175; femoral, n=175) to ensure sufficient statistical power for detecting meaningful clinical differences [9].

Intervention

Patients underwent primary PCI using either the radial or femoral access route, as decided by the interventional cardiologist based on clinical judgment and patient suitability. Standardized protocols for catheterization, management of complex coronary lesions, and stent placement were followed for both access approaches. The radial approach utilized a 6-French catheter, whereas the femoral approach used either a 6-French or 7-French sheath, depending on lesion complexity. Anticoagulation during PCI involved weight-adjusted unfractionated heparin, and all patients were on dual antiplatelet therapy comprising aspirin and a P2Y12 inhibitor.

Outcomes

The primary outcome was the incidence of access site complications, including hematomas, pseudoaneurysms, arteriovenous fistulas, and major bleeding events requiring transfusion or extended hospitalization. Secondary outcomes included procedural success, access site crossover, and length of hospital stay. Complications were defined using the Vascular Complications Consensus Document, as outlined by Mehran et al. [8], and evaluated at the time of discharge and during follow-up visits.

Data collection

Data were collected from electronic medical records, and physical assessments were conducted at predetermined intervals. Vascular complications were assessed using a validated clinical evaluation form, employing the thrombolysis in myocardial infarction (TIMI) bleeding classification to grade complication severity. Data collectors were blinded to the PCI access route to minimize bias during the evaluation.

Statistical analysis

Data analysis was performed using Statistical Product and Service Solutions (SPSS, version 26.0; IBM SPSS Statistics for Windows, Armonk, NY). Categorical variables were summarized as frequencies and percentages, while continuous variables were presented as means and standard deviations. Chi-square tests were employed for categorical comparisons, and Student’s t-tests were used to compare continuous variables between the radial and femoral groups. To adjust for potential confounding factors such as age, gender, and comorbidities, multivariate logistic regression was conducted to evaluate the independent relationship between access site and complications. Confidence intervals were set at 95%, and a p-value of <0.05 was considered statistically significant. The Bonferroni correction was applied where appropriate to account for multiple comparisons.

Ethical considerations

The study was approved by the Institutional Ethics Committee of the Army Cardiac Centre, CMH, Lahore. The Army Cardiac Centre, CMH, Lahore, Ethical Review Board issued approval Ref No: 584/IRB/ACC/2022. Written informed consent was obtained from all participants prior to enrollment, and data confidentiality was maintained throughout the study. Access to data was restricted to authorized study personnel, in compliance with ethical standards.

## Results

A total of 350 patients were enrolled in the study from January 1, 2023, to December 31, 2023, with equal distribution between the radial (n=175, 50%) and femoral (n=175, 50%) access groups. The mean age was 62.1 years (±10.2) for the radial group and 63.4 years (±11.1) for the femoral group, with no statistically significant difference (t = 1.11, p = 0.27). Of the total participants, 220 were male (62.9%), with 105 (60.0%) in the radial group and 115 (65.7%) in the femoral group (χ² = 0.86, p = 0.35). The median BMI was 28.6 kg/m² (IQR: 24.8-31.3) for the radial group and 29.1 kg/m² (IQR: 25.2-32.1) for the femoral group (t = 0.77, p = 0.44). Table 1 presents the baseline characteristics of participants, including comorbidities and medication history.

**Table 1:** Baseline characteristics of study participants Continuous variables (e.g., age and BMI) were analyzed using Student’s t-test, and categorical variables (e.g., sex and comorbidities) were analyzed using chi-square tests (χ²). Data are expressed as mean (±SD) for continuous variables and median (IQR) for non-normal variables. p-values < 0.05 indicate statistical significance.

Variable	Radial Group (n=175)	Femoral Group (n=175)	t/χ² Value	p-Value
Age (years) Mean (±SD)	62.1 (±10.2)	63.4 (±11.1)	1.11	0.27
Male, n (%)	105 (60.0)	115 (65.7)	0.86	0.35
BMI (kg/m²) Median (IQR)	28.6 (24.8–31.3)	29.1 (25.2–32.1)	0.77	0.44
Hypertension, n (%)	110 (62.9)	115 (65.7)	0.30	0.58
Diabetes Mellitus, n (%)	75 (42.9)	82 (46.9)	0.46	0.50
Dyslipidemia, n (%)	90 (51.4)	88 (50.3)	0.03	0.84
Smoking History, n (%)	70 (40.0)	80 (45.7)	1.03	0.31
Previous MI, n (%)	40 (22.9)	45 (25.7)	0.26	0.61

**Figure 1:**
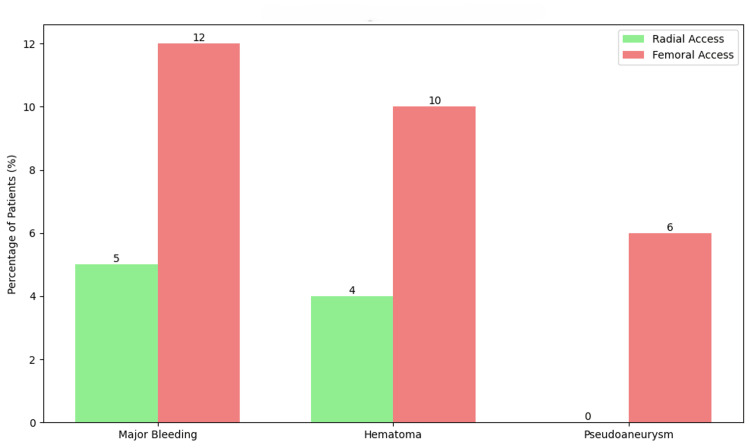
Incidence of access site complications

The incidence of access site complications was significantly lower in the radial group (20 patients, 11.4%) compared to the femoral group (40 patients, 22.9%) (χ² = 7.35, p = 0.007). Major bleeding events were also significantly reduced in the radial group (four patients, 2.3%) compared to the femoral group (15 patients, 8.6%) (χ² = 6.61, p = 0.01). Figure 1 illustrates the comparison of access site complications between the two groups.

Secondary outcomes, including procedural success, access site crossover, and length of hospital stay, are summarized in Table 2. Procedural success rates were comparable between the groups, with 170 (97.1%) successful interventions in the radial group versus 165 (94.3%) in the femoral group (χ² = 1.03, p = 0.31). The radial group exhibited a higher incidence of access site crossover (4.6%) compared to the femoral group (1.1%) (χ² = 3.88, p = 0.05). The median length of hospital stay was significantly shorter for the radial group (three days, IQR: 2-4) compared to the femoral group (five days, IQR: 4-7) (t = 8.24, p < 0.001).

**Table 2:** Secondary outcomes and procedural characteristics Chi-square tests (χ²) were used for categorical variables and t-tests for continuous variables. Hospital stay is expressed as median (IQR) due to non-normal distribution. p-values < 0.05 indicate statistical significance.

Outcome/Characteristic	Radial Group (n=175)	Femoral Group (n=175)	t/χ² Value	p-Value
Procedural Success, n (%)	170 (97.1)	165 (94.3)	1.03	0.31
Access Site Crossover, n (%)	8 (4.6)	2 (1.1)	3.88	0.05
Length of Hospital Stay (days) Median (IQR)	3 (2–4)	5 (4–7)	8.24	<0.001
Contrast Volume (mL) Mean (±SD)	180.5 (±40.7)	185.2 (±38.9)	0.77	0.44

**Figure 2:**
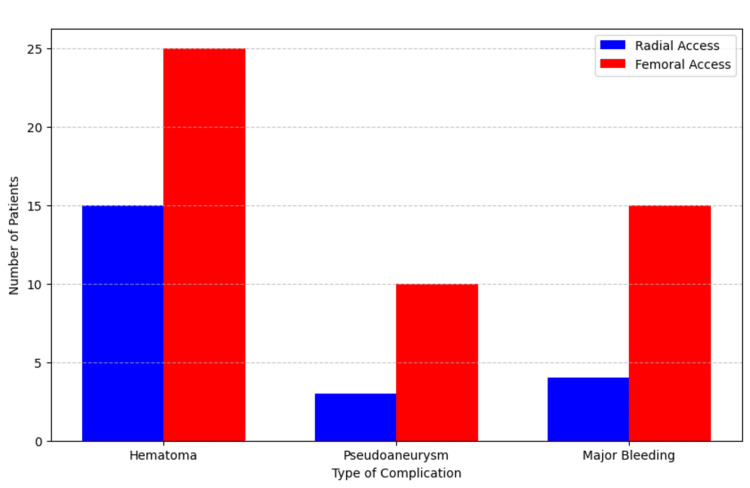
Distribution of vascular complications by access route

Unexpected findings included a higher frequency of pseudoaneurysms in the femoral group (10 patients, 5.7%) compared to the radial group (three patients, 1.7%) (p = 0.03). Minor hematomas were observed in 15 patients (8.6%) in the radial group and 25 patients (14.3%) in the femoral group, though this difference was not statistically significant (p = 0.11). Figure 2 presents the distribution of different types of vascular complications between the two groups.

This figure illustrates the distribution of various vascular complications, including pseudoaneurysms and hematomas, across the radial and femoral access groups.

Statistical analyses were performed using chi-square tests for categorical variables and Student's t-tests for continuous variables. Multivariate logistic regression analysis was conducted to adjust for potential confounders such as age, gender, BMI, and comorbidities. The regression model identified femoral access as an independent predictor of increased complications, with an odds ratio (OR) of 2.1 (95% CI: 1.4-3.4, p = 0.001). Table 3 summarizes the regression analysis for access site complications.

**Table 3:** Multivariate logistic regression analysis for access site complications This table shows the results of multivariate logistic regression analysis for factors associated with increased risk of access site complications. Odds ratios (OR) with 95% confidence intervals (CI) and corresponding p-values are provided for each variable. A P value less than 0.05 is considered significant. An OR greater than 1 indicates an increased likelihood of complications, while an OR less than 1 indicates a decreased likelihood.

Variable	Odds Ratio (OR)	95% Confidence Interval (CI)	p-Value
Femoral Access	2.1	1.4–3.4	0.001
Age (years)	1.02	0.98–1.05	0.22
Male Sex	1.3	0.8–2.1	0.25
Hypertension	1.1	0.7–1.8	0.55
Diabetes Mellitus	1.5	1.0–2.3	0.06

## Discussion

This prospective study compared access site complications between radial and femoral approaches in primary PCI for complex coronary lesions. The findings demonstrated a significantly lower rate of access site complications in the radial group (11.4%) compared to the femoral group (22.9%), including a reduced incidence of major bleeding events (2.3% vs. 8.6%) and shorter median hospital stays (three days vs. five days). These results align with previous evidence suggesting that the radial approach offers safety advantages over femoral access, particularly in reducing vascular complications [10]. The study’s focus on complex lesions provides additional insights into the optimal access site for high-risk PCI cases, addressing a gap in the existing literature.

The findings of this study are consistent with large-scale trials, such as the MATRIX and RIVAL studies, which have demonstrated that radial access significantly reduces major adverse cardiovascular events (MACE), bleeding, and mortality compared to femoral access in patients undergoing PCI for acute coronary syndromes (ACS). Both trials provide robust evidence that radial access minimizes bleeding risks and vascular complications, supporting the observed lower complication rates with radial access in this study [11,12].

Jolly et al. found that radial access significantly decreases major bleeding events compared to femoral access, reinforcing the observed reduction in the present study [10]. However, the procedural success rates in this study did not differ significantly between the groups, which contrasts with some reports, such as those by Mehta et al., who indicated a slight procedural advantage with the radial approach [8]. Variability in operator experience and patient selection may account for these differences, underscoring the need for individualized assessment when choosing the access route.

The higher access site crossover in the radial group (4.6%) compared to the femoral group (1.1%) aligns with findings from studies such as Romagnoli et al., who noted an increased likelihood of crossover due to anatomical factors such as small radial artery diameter or complex vessel tortuosity [13]. The potential for radial access failure due to anatomical challenges may still warrant femoral access in select cases, highlighting the importance of pre-procedural assessment to optimize access site selection.

The results suggest that radial access should be the preferred approach for primary PCI in complex cases, given its association with fewer complications and reduced recovery time. This could lead to shorter hospital stays and lower healthcare costs, supporting the broader adoption of the radial approach in interventional cardiology [5]. Additionally, the use of radial access is consistent with evolving guidelines that recommend minimizing bleeding risks during PCI, especially in high-risk patients [14].

Future multicenter studies with diverse patient populations could provide a more comprehensive understanding of access site-related outcomes in complex PCI [15]. Investigating strategies to reduce radial access crossover, such as routine use of pre-procedural imaging to evaluate vessel suitability, could also enhance clinical decision-making. Additional research is warranted to further explore the benefits of radial access in different subsets of complex PCI cases, particularly in patients requiring large-bore catheters or with challenging anatomical features [16,17].

Limitations

Several limitations of the study should be noted. The single-center design may limit the generalizability of the findings to other populations. Furthermore, the exclusion of patients with significant comorbidities or prior coronary artery bypass grafting may restrict the applicability of the results to broader patient cohorts. Finally, operator experience and variability in procedural techniques could have influenced the outcomes.

## Conclusions

This study indicates that the radial approach is associated with fewer complications compared to the femoral approach in primary PCI for complex coronary lesions. The findings support the use of radial access as the preferred choice, where feasible, due to its lower rates of access site complications, major bleeding, and shorter hospital stays. These results have significant implications for clinical practice, suggesting that adopting a "radial-first" strategy in complex PCI could improve patient outcomes and reduce healthcare costs. Further research, especially multicenter trials involving diverse patient populations, is necessary to validate these findings and optimize access site selection strategies for high-risk PCI procedures.
